# Principles of Human Physiology

**Published:** 1855-11

**Authors:** 


ART. V.—*Principles of Human Physiology, with their chief applications to Psychology, Pathology, Therapeutics, Hygiène, and Forensic Medicine.* By WILLIAM B. CARPENTER, M. D., F. R. S., etc., etc. A new American from the last London edition, with 261 Illustrations. Edited with additions by FRANCIS GURNEY SMITH, M. D., Professor of the Institutes of Medicine in the Medical Department of Pennsylvania College, etc. Philadelphia: Blanchard & Lea. 1855. pp. 902. 8vo.

---

Dr. Carpenter stands confessedly at the head of English authors on Physiology. No work in our language will compare with his several productions on this subject. He is not only the most original of our writers, but the most comprehensive, and most philosophical. If he is exceeded in extent of original research by German and French physiologists, by none of them is he equalled as a clear, logical exponent of the facts and principles of the science. His "Human Physiology" had grown to be so voluminous that he has wisely decided to transfer from it to his "General Physiology" such parts as belong properly to the latter work.

The "overgrown bulk" of the last edition has therefore disappeared in this, from which is excluded the summary of Animal Chemistry, and of the structure and actions of the Animal Tissues, amounting in all to about 240 pages. On the other hand some valuble additions have been made to the chapters on the Organic Functions, and many portions have been re-written, so that his treatise represents the convictions and opinions of the author as completely as if it were making its appearance for the first time. Dr. Carpenter is not ashamed to avow that he has changed his opinions on several subjects since his work was first written. In the rapid progress of physiological science it would be weakness in an author to pretend that his opinions suffered no change. The whole subject of the science has been changed within the last half of the present century, and writers who would not become obsolete must modify their views in accordance with this revolution. Dr. Carpenter is to be commended on another account—he is ready to welcome truth from whatever quarter it may come, and to give the credit due to every investigator. He has repeatedly made honorable mention of Americans who have contributed facts to the science or suggested plausible theories in regard to any of its phenomena. The present edition contains a summary of Dr. Dalton's researches on the distinctions between the corpus luteum of simple menstruation and that of pregnancy.

Concerning certain states and actions of the cerebrum Dr. Carpenter has put forth views in the successive editions of his work which were received at first with a large measure of skepticism, but which are now gaining the general assent of physiologists. This organ, the instrument of the mind, it cannot be questioned, is capable of acting without the consciousness of the individual. The brain, through which we think, may be active and yet excite no conscious thought. This process Dr. C. terms "unconscious cerebration." "It would have been scarcely possible," he remarks, "to conceive of any more opposite and convincing proof of that independent automatic activity of the cerebrum and of its involuntary influence in producing muscular contraction, than that which was afforded by the epidemic of 'Table-turning and Table-talking,' which, originating in the United States, began to spread through Europe just at the epoch of the publication of the former edition, in which that doctrine was first distinctly developed." It would be superfluous to recommend a work which has acquired so wide a popularity.

